# ZNF580 – a brake on Interleukin-6

**DOI:** 10.1186/s12950-018-0196-5

**Published:** 2018-10-22

**Authors:** Philipp Stenzel, Kaj Nagorsen, Jonathan Bernd, Ulrike Leppert, Andreas Zakrzewicz, Janine Berkholz

**Affiliations:** 1 Institute of Physiology, Charité - Universitätsmedizin Berlin, corporate member of Freie Universität Berlin, Humboldt-Universität zu Berlin, and Berlin Insitute of Health, Virchowweg 6/ CCO, Charitéplatz 1, 10117 Berlin, Germany; 2 Institute of Pathology, Universitätsmedizin Mainz, Mainz, Germany

**Keywords:** ZNF580, Interleukin 6, Monocytes, LPS, Sepsis

## Abstract

**Background:**

Zinc finger protein 580 (ZNF580) was reported to modulate angiogenesis, endothelial homeostasis and blood pressure control. ZNF580 regulated genes include VEGF-A and IL-8. However, it is unknown if ZNF580 could play a role during inflammation. The aim of this study was to find out if ZNF580 affects the expression of IL-6, if it occurs in monocytic cells and responds to inflammatory mediators.

**Results:**

Overexpression of ZNF580 reduced LPS-induced promotor activity of IL-6. Consistently, overexpression of ZNF580 reduced by half the LPS-induced expression of IL-6. ZNF580 was strongly expressed in the nucleus of MonoMac6, a human monocytic cell line. LPS-stimulated IL-6 secretion increased when ZNF580 was suppressed with siRNA. After stimulation of MonoMac6 with LPS for 24 h, ZNF580 negatively correlated with the amount of secreted IL-6. In response to LPS, ZNF580 was increased within the first 8 h, followed by a marked decrease after 16 h. This decrease coincided with sustained IL-6 production.

**Conclusion:**

This study demonstrated that ZNF580 inhibits LPS-induced expression of IL-6. ZNF580 was highly expressed in monocytic cells and therefore may contribute to the modulation of its IL-6 production, at least in response to LPS. This suggests cooperation between ZNF580 and NFκB, which could play a role during sepsis.

## Background

Zinc finger protein 580 (ZNF580) belongs to the family of zinc finger proteins of the cystidin-2 histidine-2 type (C2H2) [1] and contributes to the expression of vascular endothelial growth factor A (VEGF-A), matrix metallopeptidase 2 (MMP2) and Mothers against decapentaplegic homolog 2 / SMAD family member 2 (SMAD2) in endothelial cells [2, 3]. Via these molecules, ZNF580 is presumably involved in the regulation of angiogenesis, endothelial cell function and endothelial nitric oxide synthase (eNOS) expression in response to transforming growth factor β (TGF-β) [2–4]. Endothelial cell migration is increased after gene transfer of ZNF580 [5–7].

In addition, ZNF580 is involved in endothelial inflammation [8, 9]. Stimulation of human umbilical vein endothelial cells (HUVEC) with native low-density lipoprotein (LDL) increases ZNF580 mRNA expression in a dose-dependent manner while its knockdown causes enhanced production of interleukin 8 (IL-8). Accordingly, the production of IL-8 is enhanced with increasing portions of oxidized LDL (oxLDL), because these are associated with a decrease of ZNF580 mRNA [8]. In contrast, the increase of ZNF580 in EA.hy926 by hydrogen peroxide (H2O2) was reported to induce the production of IL-8 via nuclear factor kappa light chain enhancers of activated B cells (NFκB) [9].

By now, the role of ZNF580 in the regulation of cytokines and inflammation is an object of investigation. Since, all relevant studies so far have been conducted in endothelial cells, little is known how ZNF580 contributes to inflammatory processes in other cell types. Therefore, the purpose of this study was to elucidate the role of ZNF580 in the inflammatory reaction of the human leukemia derived monocytic cell line MonoMac6 [10], a model cell line for monocytes.

Monocytes belong to the innate immune system and are pivotal regulators of inflammation. They mediate inflammatory responses via the generation of cytokines and reactive oxygen species (ROS) as defence mechanisms especially against bacterial infections [11, 12]. One of the most potent activators of monocytes is the cell wall component lipopolysaccharide (LPS) of gram-negative bacteria [13]. Amongst others, the cytokine interleukin 6 (IL-6) is secreted by monocytes in response to LPS. IL-6 is a key mediator of the acute phase response in sepsis [14] and is widely used as a marker in clinical routine [15, 16].

The aim of this study was to investigate whether ZNF580 can modulate the expression of IL-6, whether it is expressed in monocytic cells and in turn responds to inflammatory mediators.

## Methods

### Cell culture

MonoMac6 cells were cultivated in RPMI-1640-Medium supplemented with 10% (*v*/v) fetal calf serum (FCS), 2 mM L-Glutamine,and 0,5% (v/v) penicillin/streptomycin (PEST) (all purchased from Biochrom, Berlin, Germany). Cells were cultured in a humidified atmosphere containing 5% CO2 at 37 °C and were passaged every two to three days. For the transfection of cells with small interfering ribonucleic acids (siRNAs) OPI-supplement (Sigma-Aldrich, München, Germany) was added to the cell culture medium.

### Antibodies

The following antibodies were used to detect the respective proteins: primary antibodies against ZNF580 (ARP39236_P050, Aviva Bio Systems, San Diego, CA, USA; immunocytochemistry 1:100, immunoblotting 1:500), and β-Actin (A-5441, Sigma Aldrich, München, Germany; 1:2.000); secondary goat-anti-rabbit antibodies to detect ZNF580 (P0448, Dako, Glostrup, Denmark; 1:5.000); secondary rabbit-anti-mouse antibodies to detect β-Actin (P0260; Dako, Glostrup, Denmark; 1:20.000), and Cy-3-conjugated secondary antibodies for fluorescence staining of ZNF580 (TM 111–165-045, Jackson Immunoresearch Laboratories, West Grove, PA, USA; 1:1.000).

### Fluorescence microscopy of ZNF580

Fluorescence microscopy was performed as described before [7]. Cells were fixed with ice cold methanol. Nuclei were stained with 4′,6-diamidino-2-phenylindole dihydrochloride (DAPI) (Merck, Darmstadt, Germany; 1:10.000). Cells were analysed with a Zeiss Axioskop 40 microscope and a Zeiss object lens Neofluar, 16/0.4 (Carl Zeiss, Jena, Germany). The images were documented via a digital camera (Kappa, DX4, Kappa, Gleichen, Germany) and the overlay done with Image J.

### Protein extraction and quantification

Cells were harvested, washed with ice-cold phosphate buffered saline (PBS) and centrifuged. For whole cell lysates, the pellet was resuspended in extraction buffer (20 mM NaPi (pH 7.8), 1% (*v*/v) Triton-100, 150 mM NaCl, 2.5 mM EDTA, 1 mM Na_3_VO_4_, 50 mM NaF, 1 mM PMSF, 0.02 μg/μL leupeptin, 0.02 μg/μL pepstatin A, 0.02 μg/μL aprotinin, Sigma-Aldrich, München, Germany) and subsequently centrifuged. Protein concentration was determined according to the Bradford method.

### Immunoblotting

To reduce any possible remaining disulphide bonds, DTT was added at 100 mM (20 mM final) to Laemmli buffer (i.e. sample buffer). 30 μg of protein lysate was run in each lane on a 12% acrylamide gel. Protein was blotted on a polyvinylidene difluoride (PVDF) membrane by wet transfer. The membrane was blocked with 5% milk powder in phosphate buffered saline-Tween (PBS-T) followed by incubation with primary antibodies over night at 4 °C. Afterwards the membrane was washed with PBS-T three times and incubated at room temperature with specific horseradish peroxydase-coupled secondary antibodies. Protein detection was done using the Western Lightning chemiluminescence kit (Perkin Elmer, Waltham, Massachusetts, USA) and exposure to Hyperfilm enhanced chemiluminescence (ECL) (Amersham, Freiburg, Germany) following the manufacturer’s instructions. Densitometric quantification was performed with the software OneD-Scan (Scanalytics, Rockville, MD, USA).

### Transfection with ZNF580 expression vector

For transient transfection of full-length ZNF580, the human expression plasmid pcDNA3.1^+^/C-(K)-DYK-ZNF580 (GenScript, USA Inc.) was used in combination with the TurboFect reagent (Thermo Scientific) according to the manufacturer’s instructions.

### Reporter gene assay

HEK 293 cells were transiently transfected with 0.5 μg of human ZNF580 expression plasmid (GenScript, USA Inc.), by which the mRNA level of ZNF580 was increased more than 80 fold (not shown), and 0.5 μg pBABE lucIL6 reporter using TurboFect transfection reagent (Thermo Scientific) according to the manufacturer’s instructions. pBABE lucIL6 was a kind gift from Sheila Stewart (Addgene plasmid # 52884) [17]. The vector contains the human IL-6 promoter region upstream of the luciferase-encoding sequence of plasmid pBABE hygro. Six hours after transfection cells were either stimulated with LPS (1 μg/mL) for 24 h or remained unstimulated. The cells were harvest in lysis buffer and assayed for firefly activity as described by the manufacturer of the Luciferase Assay System (Promega, Inc). The amount of luciferase activity in each sample was quantified by a Varioskan™ Flash Multimode Reader (Thermo Scientific).

### Cell stimulation experiments

For stimulation experiments MonoMac6 were treated with LPS (O55:B5; Sigma Aldrich, Germany, final concentrations as indicated), fMLP 100 nmol/L (Sigma Aldrich, München, Germany) or TNF-α 10 ng/mL (R&D Systems, Minneapolis, MN, USA), all dissolved in PBS or dimethyl sulfoxide (DMSO). Vehicle alone served as a negative control. Cells were harvested after different times of stimulation as indicated and used for either mRNA or protein analysis, respectively. Supernatants were collected for IL-6 quantification. Time-dependent effects were analysed following stimulation with LPS 10 ng/mL final concentration.

### RNA isolation and reverse transcription

Total RNA was extracted from MonoMac6 cells using the RNeasy Kit (Qiagen, Hilden, Germany) according to the manufacturer’s instructions. mRNA of 2 μg total RNA was transcribed into complimentary DNA (cDNA) using a reverse transcriptase kit (M1701, Promega, Madison, WI, USA).

### Real-time PCR

Specific primers used for amplification of ZNF580, glyceraldehyde 3-phosphate dehydrogenase (GAPDH) and IL-6 are shown in table 1and were purchased from Eurofins, Hamburg, Germany. Real-time PCR was performed with a Rotorgene 2000 (Corbett Life Science, Mortlake, NSW, Australia) using the SYBR-green master mix (QuantiTect SYBR Green PCR Kit, Qiagen, Hilden, Germany). The copy numbers were calculated by standard curves which were generated using PCR-produced standard templates (the respective longer products in Table 1). Copy numbers of ZNF580 and IL-6 were normalised to copy numbers of GAPDH. The quality of amplificons was checked via melting curves. For all real-time PCR analyses the corresponding non-template and non-reverse transcription (RT) controls were done.

**Table 1:** Primer sequences, product sizes and annealing temperatures used to amplify the corresponding cDNA templates. The respective longer products were used to generate standard amplification curves as described in the methods section

Templates	Forward Primer	Reverse Primer	Product size	Annealing Temperature
ZNF580	5’ CCC TCC ACT CCT TCC T 3’	5’ ACG TGA AGG GCT TGA GGT C 3’	262 bp	60 °C
ZNF580	5’ CGC CAC CTC CTC ATC GAC GC 3’	5’ TCC GGG CAG CTG TAG CCC TT 3’	134 bp	60 °C
GAPDH	5′ TGG TGG ACC TGA CCT GCC GTC 3’	5′ AGG GGT CTA CAT GGC AAC TG 3’	415 bp	60 °C
GAPDH	5’ TCA AGA AGG TGG TGA AGC AG 3’	5’ CCC TGT TGC TGT AGC CAA AT 3’	198 bp	60 °C
IL-6	5’TGC AAT AAC CAC CCC TGA CC 3’	5′ GTG CCC ATG CTA CAT TTG CC 3’	163 bp	58 °C
IL-6	5’ TGC CAG CCT GCT GAC GAA G 3’	5’ AGC TGC GCA GAA TGA GAT GAG 3’	77 bp	58 °C

### Transfection of MonoMac6 with small interfering RNA (siRNA)

Transfection of MonoMac6 cells was performed using specific siRNAs against ZNF580 (Thermo Fisher Scientific/Invitrogen, Waltham, MA, USA), scrambled siRNA (Thermo Fisher Scientific/Ambion, Waltham, MA, USA) as negative control and Cy3-labelled siRNA against GAPDH (Thermo Fisher Scientific/Ambion, Waltham, MA, USA) as a positive control and to determine the transfection rates. The empirically determined optimal concentration of siRNA was 50 nM to 70 nM. The transfection reagent was Lipofectamine 2000 (Thermo Fisher Scientific / Invitrogen, Waltham, MA, USA), which was used according to the manufacturer’s protocol. Knockdown of the target mRNA was monitored 24 h after transfection of siRNA by real-time PCR and immunoblotting. For LPS stimulation MonoMac6 cells were transfected with 70 nM scrambled siRNA or ZNF580-specific siRNA, respectively, for 24 h. Thereafter, cells were pelleted and resuspended in RPMI 1640 medium with supplements. LPS dissolved in PBS was added to the cells in a final concentration of 10 ng/mL for times as indicated.

### IL-6 protein measurement in cell culture supernatants

IL-6 protein was determined in cell culture supernatants using the Quantikine Elisa Kit for human IL-6 (R&D Systems, Minneapolis, MN, USA) according to the manufacturer’s protocol. Samples were analysed in triplicates. IL-6-production rate was calculated with the formula, whereby T2 and T1 were two adjacent time points: (IL-6_T2_-IL-6_T1_)/(T_2_-T_1_).

### Statistical analysis

Data are given as mean ± standard error. All experiments were independently repeated at least three times. Statistical significance of cell treatments in comparison to untreated controls was calculated by Student’s *t*-test. To analyse the linear correlation, Pearson’s correlation coefficient r was calculated. The error probability *p* ≤ 0.05 was considered significant for all statistical tests used.

## Results

### Repression of the interleukin 6 gene promotor by ZNF580

**Fig. 1:**
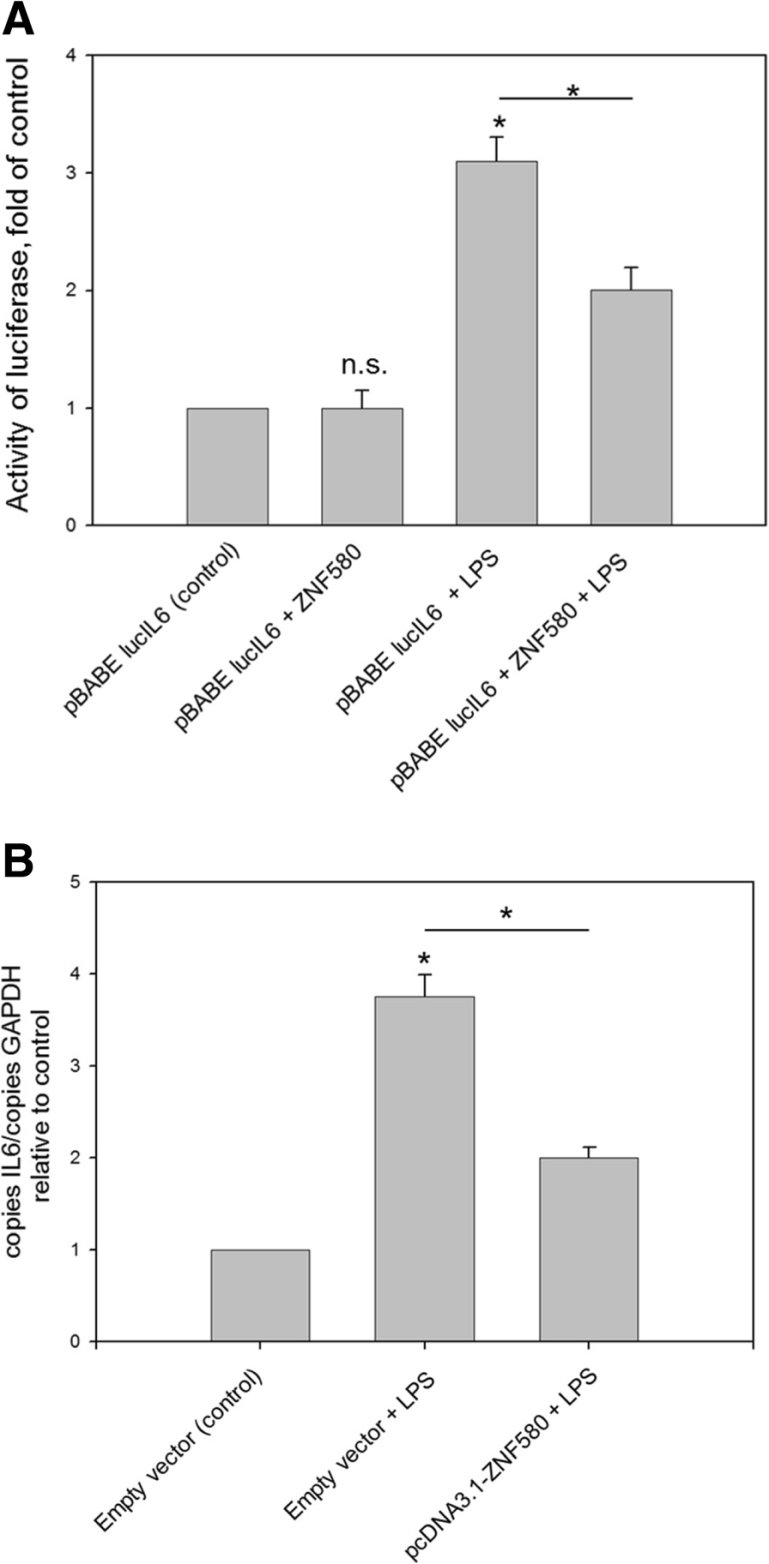
ZNF580 decreased the activity of the IL-6 promotor and the mRNA transcript level. **a** HEK 293 cells were transiently transfected with 0.5 μg human ZNF580 expression plasmid (GenScript, USA Inc.) and 0.5 μg pBABE lucIL6 reporter using TurboFect transfection reagent (Thermo Scientific). pBABE lucIL6 was a gift from Sheila Stewart (Addgene plasmid # 52884). Six hours after transfection cells were either stimulated with LPS (1 μg/mL) for 24 h or remained unstimulated. The cells were harvested and analysed for luciferase activity (**a**). Measurements were done in duplicates, results are given as mean ± SE, of *n* = 6 independent experiments. **b** HEK 293 cells were transfected with ZNF580 expression vector or control empty vector. Transfected cells were stimulated with 1 μg/mL LPS for 24 H*. IL*-6 mRNA was quantified by real time PCR and normalised to the corresponding GAPDH values (**b**). Results are given as mean ± SEM, of *n* = 3 independent experiments. *: *p* ≤ 0,05

The effect of human ZNF580 on IL-6 promotor activity was evaluated in transient transfection experiments in which the human pBABE lucIL6 reporter construct was cotransfected with or without the human ZNF580 wildtype gene into HEK293 cells 6 h before stimulation with LPS. IL-6 transcription was not changed by cotransfection of ZNF580 but remained on its basal level. LPS (1 μg/mL, 24 h) strongly induced the promotor activity of IL-6, as expected. However, cotransfection of ZNF580 significantly reduced LPS-dependent induction of the IL-6 promoter to about half (Fig. 1a).

### ZNF580 decreased LPS-induced expression of IL-6

As the activity of the IL-6 promoter is reduced by ZNF580, we wanted to examine as a next step whether ZNF580 also reduces the expression of IL-6. For this purpose, ZNF580 was overexpressed in HEK293 cells, halving LPS-induced expression of IL-6 (Fig. 1b).

### ZNF580 was expressed in MonoMac6 cells and its expression was altered by LPS

**Fig. 2:**
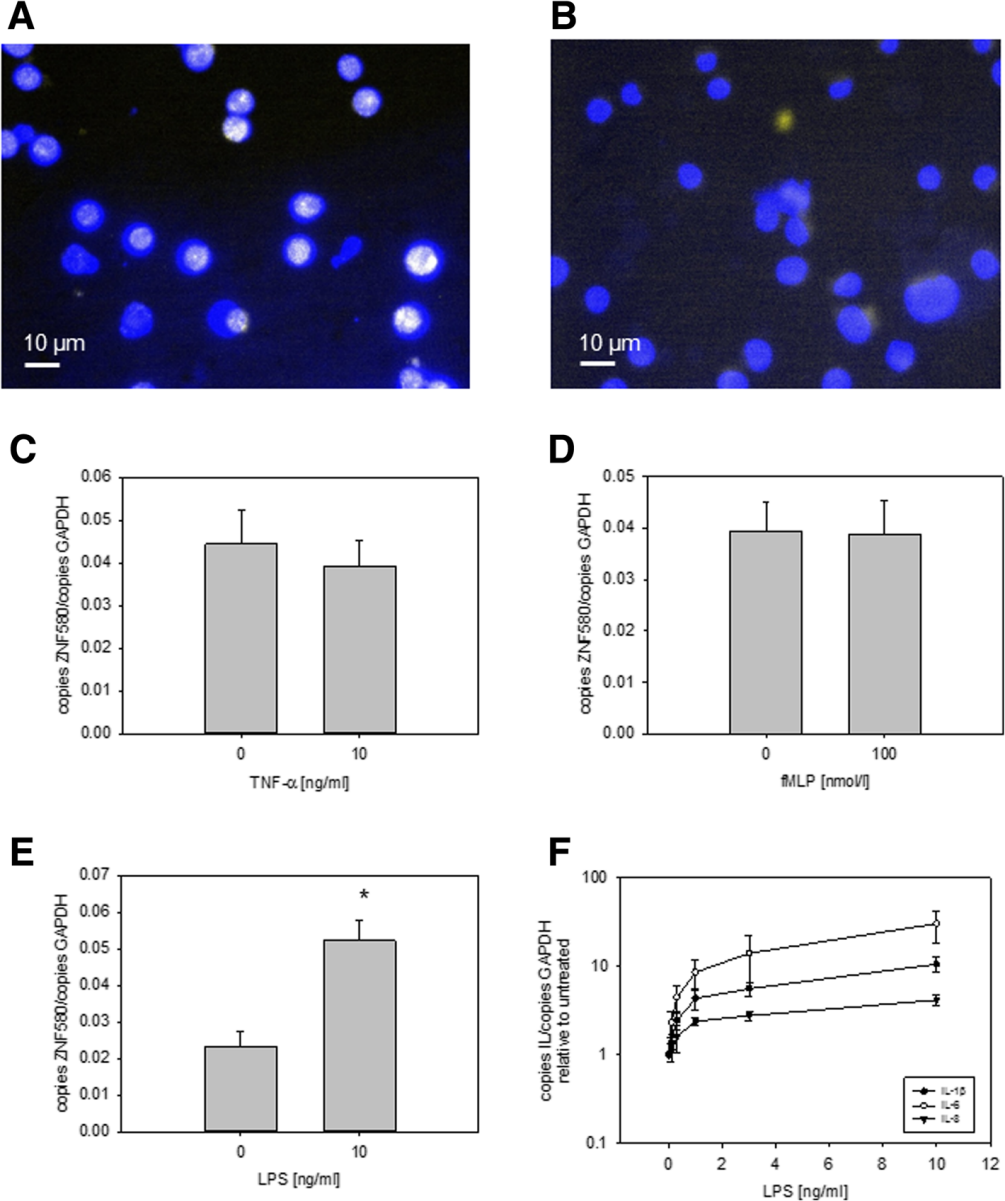
ZNF580 in MonoMac6. Immunofluorescence staining of ZNF580 in MonoMac6 cells (yellow signal) and counterstaining of nuclei (blue signal) revealed an overlap of both signals (**a**). In the negative control, no other signal was detectable than DAPI (**b**). 1 × 10^6^ cells were treated for 24 h with TNF-α (10 ng/mL) (**c**), fMLP (100 nmol/L) (**d**) and LPS (10 ng/mL) (**e**). As a control (0 nmol/L; 0 ng/mL) the vehicle PBS was used. ZNF580 mRNA was measured by real time PCR and expression normalised to corresponding GAPDH-values. After 24 h ZNF580 mRNA was unchanged by TNFα (**c**) and fMLP (**d**) but increased after treatment of MonoMac6 with LPS (**e**). The mRNA expression of IL-6, IL-8 and IL-1β were analysed via real-time-RT-PCR after treatment of 2 × 10^6^ cells with different concentrations of LPS (0; 0.1; 0.3; 1, 3, or 10 ng/mL, respectively) for 24 h (**f**). All results are shown as mean ± SEM, *n* = 3, *: *p* ≤ 0,05

To determine the expression and localization of ZNF 580 in monocytic cells, MonoMac6 cells were cultivated and subsequently analysed. Immunofluorescent staining revealed that ZNF580 is expressed in this monocytic cell line and localized intracellularly almost exclusively in the nuclear region (Fig. 2a). Controls without the primary antibody did not show any staining (Fig. 2b). We sought for a physiologic stimulus of inflammation, which was able to influence ZNF580 expression. Therefore, MonoMac6 were stimulated with 10 ng/mL TNF-α (Fig. 2c), 100 nM fMLP (Fig. 2d) or 10 ng/mL LPS (Fig. 2e) for 24 h. Under these conditions ZNF580 mRNA was significantly increased through LPS treatment, whereas neither fMLP nor TNF-α showed any effect. Expression of IL-1β, IL-6 and IL-8 mRNA was dose-dependently increased in response to LPS (Fig. 2f). IL-6 mRNA showed the highest increase (Fig. 2f).

### RNAi knockdown of ZNF580 increased LPS-induced IL-6 production

**Fig. 3:**
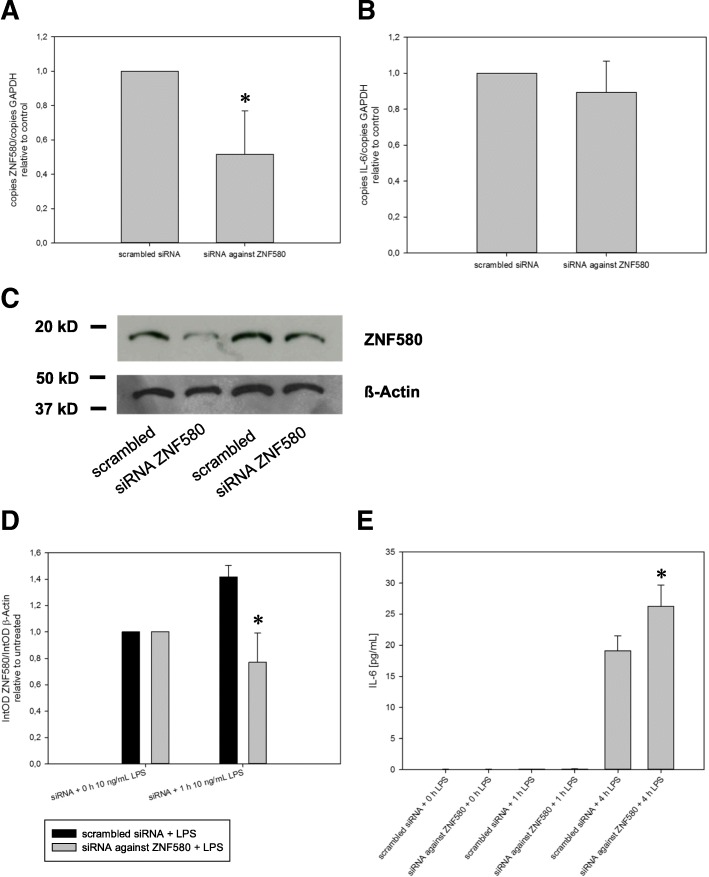
Increased IL-6-production after ZNF580-knockdown. Unstimulated MonoMac6 cells were transfected with scrambled siRNA or specific siRNA against ZNF580 mRNA. There was a significant knockdown of ZNF580 mRNA (**a**) and protein (**c**) at 24 h, which did not alter basal IL-6 expression (**b**). Transfected MonoMac6 were stimulated with 10 ng/mL LPS. The LPS-induced increase in ZNF580 was abolished when cells were transfected with siRNA against ZNF580 (**d**). LPS-induced IL-6 secretion was increased when the increase of ZNF580 was blocked by siRNA (**e**). IL-6 and ZNF580 mRNA were quantified by real-time-PCR and normalised to the corresponding GAPDH values. ZNF580 protein was analysed by immunoblotting and normalised to the corresponding β-Actin values. IL-6-protein in the supernatant was measured by ELISA. All results are shown as mean ± SEM, *n* = 3, *: *p* ≤ 0,05 (siRNA against ZNF580 versus scrambled siRNA of the respective group)

MonoMac6 cells were transfected with specific siRNA against ZNF580 or scrambled siRNA as a negative control. These samples were then analysed for ZNF580 mRNA (A) and protein (C). Thus, as shown in Fig. 3a/c, we were able to downregulate ZNF580 transcript levels by about 50% in these cells. By contrast, an unspecific (“scrambled”) control siRNA had no effect. IL-6 mRNA was also determined by real time PCR (Fig. 3b). This demonstrated that knockdown of ZNF580 did not alter basal expression of IL-6. MonoMac6 were again transfected with specific siRNA against ZNF580 or scrambled siRNA as a negative control and stimulated with 10 ng/mL LPS (Fig. 3d). This showed that the LPS-induced increase in the protein level of ZNF580 was successfully abrogated by the specific siRNA treatment. Furthermore, there was a significant increase in LPS-stimulated IL-6 secretion when ZNF580 was knocked down (Fig. 3e).

### Negative correlation occurred between ZNF580 and IL-6 after 24 h of dose-dependent LPS stimulation

**Fig. 4:**
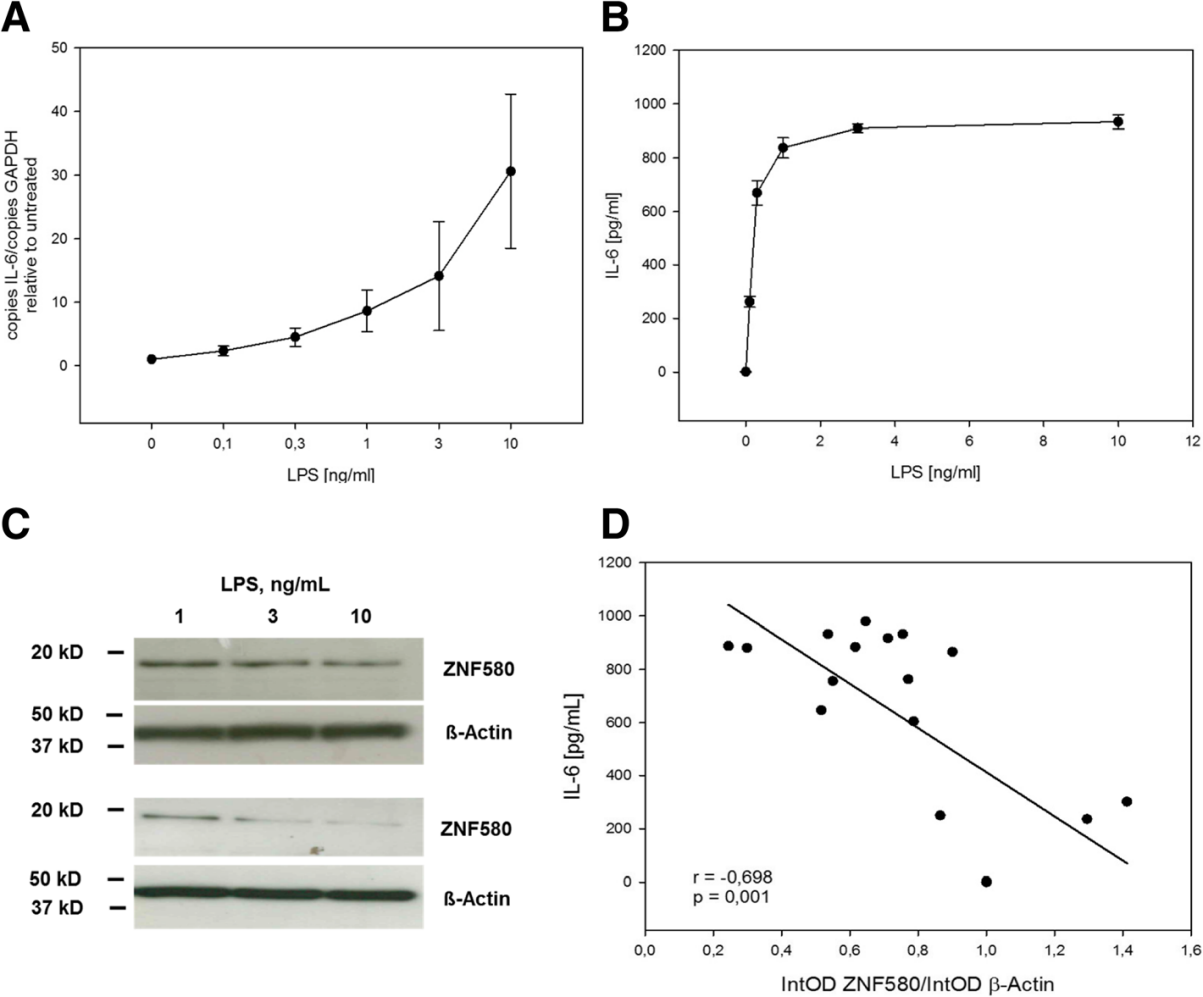
Negative correlation between ZNF580 and IL-6 after dose-dependent LPS-stimulation. MonoMac6 cells were stimulated with different LPS concentrations ranging from 0.1 to 10 ng/mL for 24 H*. IL*-6 mRNA (**a**), quantified by real-time-PCR and normalised to corresponding GAPDH-values, and protein in the supernatant (**b**), measured by ELISA, were dose-dependently increased (shown as mean ± SEM, *n* = 3). ZNF580 protein was analysed by immunoblotting (**c**). IL-6 secretion was measured over 24 h (**b** and **d**). ZNF580 was determined at the end of these 24 h and normalised to β-actin values (**d**). Under these conditions IL-6 and ZNF580 were inversely correlated (**d**). For each of four different concentrations of LPS three different samples were analysed and four untreated samples were analysed

MonoMac6 cells were incubated with different concentrations of LPS ranging from 0 ng/mL to 10 ng/mL for 24 h*. *IL-6 mRNA was determined by real time PCR, and IL-6 protein in the corresponding cell culture supernatants by ELISA. This LPS treatment showed a dose dependent increase of IL-6, both on mRNA (Fig. 4a) and protein level (Fig. 4b). The amount of ZNF580 protein was analysed in whole cell lysates by immunoblotting, a ZNF580 band was typically detected at about 18 kDa. Surprisingly, after 24 h of incubation with LPS, this band was reduced (Fig. 4c). In the cells whose IL-6 secretion was measured as a function of LPS concentration, the ZNF580 protein was also determined. This showed an inverse correlation: The more secreted IL-6 for 24 h, the lower ZNF580 was at the end (Fig. 4d). A correlation coefficient of *r* = − 0.698 (*p* = 0.001) was calculated with respect to the hypothesized line of correlation.

### ZNF580 showed a biphasic response following LPS-stimulation

**Fig. 5:**
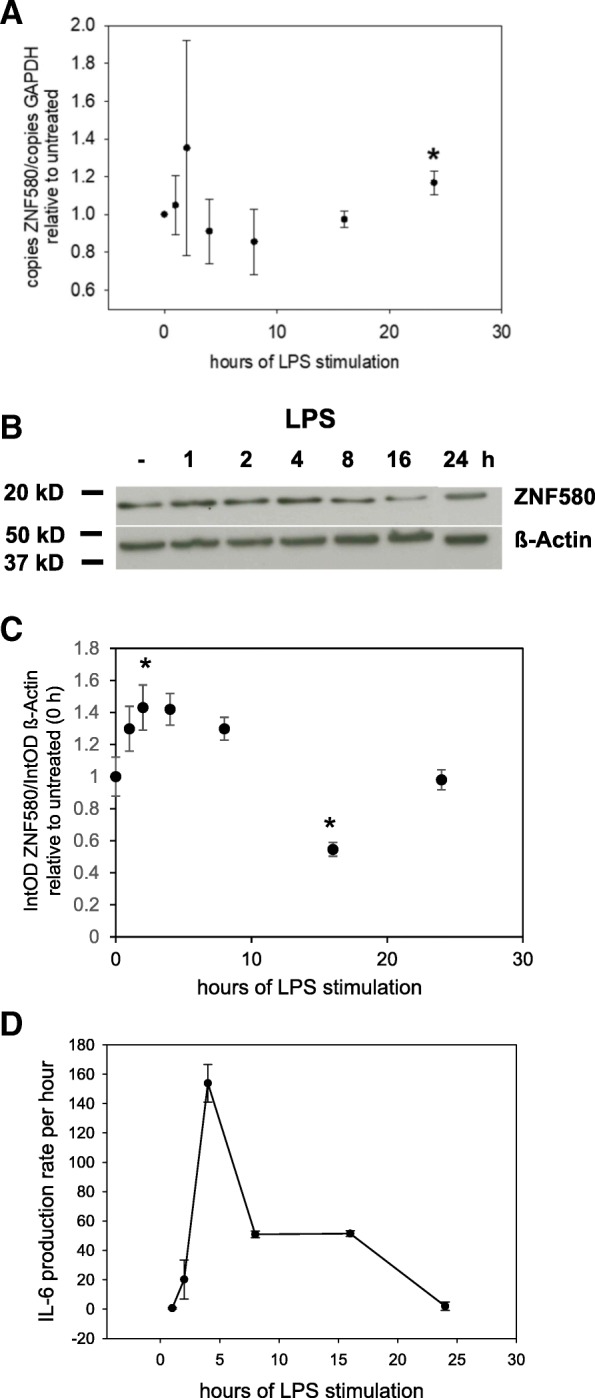
Time course of ZNF580 and IL-6-production rate after LPS treatment. MonoMac6 cells were treated with 10 ng/mL LPS over 1 h to 24 h as indicated. The cells were analysed for ZNF580 mRNA by real-time PCR at seven different time points (**a**), (*n* = 3). ZNF580 protein, analysed by immunoblotting, showed an initial increase from 1 h to 8 h and a marked decrease after 16 h, representative immunoblot (**b**) and densitometric quantification of four independent immunoblots (**c**), (*n* = 4). The IL-6 production rate determined by ELISA was calculated as the difference of secreted IL-6 protein at two adjacent time points (**d**), (*n* = 3). Results are given as mean ± SEM, *: *p* ≤ 0,05

While ZNF580 was increased by LPS following 1 h of stimulation (Fig. 3d), it was decreased following 24 h of stimulation (Fig. 4c). Because of this apparent contradiction, we have examined in more detail the time course of LPS-dependent effects on ZNF580. To achieve this, MonoMac6 cells were stimulated with LPS 10 ng / ml for 0–24 h as indicated, and then the ZNF580 mRNA (Fig. 5a) and the protein (5B / C) level were analysed. The mRNA level increased within the first two hours of LPS treatment, followed by a four and eight hour drop, which returned to control levels after 16 h and increased again after 24 h. Independent samples showed the same regulation at the protein level. Compared to the mRNA level, the protein response was somewhat delayed. Immunoblotting of samples from four independent time courses revealed a moderate increase of ZNF580 from 1 h to 8 h after LPS stimulation. This was followed by a sharp decline at 16 h. After that, ZNF580 increased again to near control values after 24 h. -IL-6 secretion rate was calculated using IL-6 concentrations from corresponding cell culture supernatants. It showed a biphasic curve with an initial peak at 4 h followed by a smaller, sustained IL-6 production. This second phase coincided with decreased levels of ZNF580 protein (Fig. 5d).

## Discussion

**Fig. 6:**
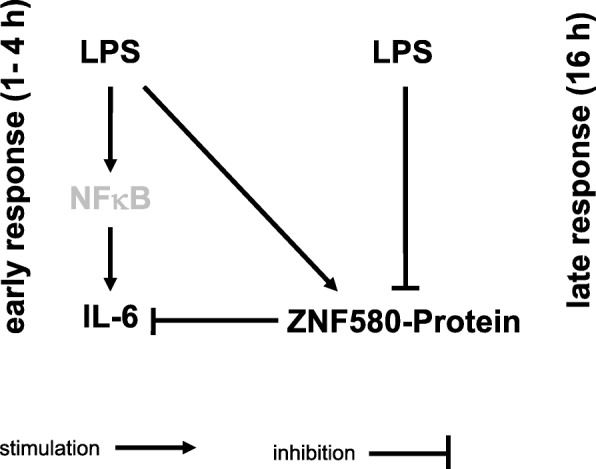
Flowchart of the hypothesised intracellular pathway after LPS stimulation of MonoMac6 cells. Stimulation with LPS led to a rapid increase in the expression and secretion of IL-6. Since ZNF580 was elevated during this time, induction of IL-6 must be via other factors, e. g. NFκB. The early increase of ZNF580 could temporally limit the initial transient peak of IL-6 production. Since ZNF580 showed a biphasic response to LPS, its decrease later supports continued production of IL-6

This study demonstrated decreased LPS-induced production of IL-6 by ZNF580. Therefore, ZNF580 should be considered as a new suppressor of the IL-6 gene. We were able to demonstrate the expression of ZNF580 in MonoMac6 cells. In these cells, ZNF580 showed a biphasic response to LPS, thus interfering with LPS-induced IL-6 production. This could lead to the distinction of an early and a late phase of LPS-induced IL-6 production (Fig. 6).

MonoMac6, an established monocytic cell line, was used as a model for monocytes [10]. Cells remained vital while treated with LPS. Cytokine production (IL-1β, IL-6, IL-8) was increased by LPS in a dose-dependent manner. The maximal effect was already achieved at about 3 ng/mL LPS. IL-6 production was elevated within relatively short times and peaked at about 4 h after LPS stimulation. This rapid reaction to even low concentrations of LPS characterized MonoMac6 as a well suited model of monocytes. The advantage of this cell line is its high responsiveness to LPS. However, there are disadvantages too. These cells are difficult to culture since they sometimes become reactive even under sterile conditions. This may increase the distribution of experimental results measured from these cells, especially between different series of experiments. Moreover, in transfection experiments with siRNA, the transfection rate is always 100% (by uptake of fluorescently labelled siRNA). Unfortunately, this does not reliably knockdown the targeted mRNA. Therefore, the effect in each sample must first be measured and then correlated with downstream effects.

While the expression of ZNF580 in endothelial cells is well documented [1–9], this study is the first demonstrating its expression in monocytes. The superposition of fluorescence signals for ZNF580 and DAPI revealed that ZNF580, like endothelial cells [8, 9], is located mainly in the nucleus of MonoMac6 cells (Fig. 2a). This localization is a prerequisite for a role as a DNA-binding factor in gene expression regulation. Likewise, ZNF580 mRNA was detectable in MonoMac6 by specific RT-PCR (Fig. 3a for instance). Immunoblotting of ZNF580 revealed a prominent and specific protein band at the expected size of approximately 18 kDA (Fig. 3c, 4c, 5b). Taken together, the expression of ZNF580 in MonoMac6 confirmed with three independent methods can be considered safe.

Using HEK 293 cells for transient overexpression of ZNF580 it was demonstrated by means of a reporter gene assay, that LPS-induced activity of the IL-6 promotor was reduced by ZNF580 (Fig. 1a). However, the basal activity of the promoter was not altered by ZNF580. Thus, ZNF580 must interfere with other transcription factors, thereby inhibiting the induction of IL-6. In addition, overexpression of ZNF580 reduced LPS-induced expression of IL-6 (Fig. 1b). This supported the concept that ZNF580 is a suppressor of IL-6 expression.

When ZNF580 was knocked down in MonoMac6 cells (Fig. 3a, c), its LPS-inducible increase was abrogated (Fig. 3d) and LPS-induced secretion of IL-6 was increased (Fig. 4e). Thus, the siRNA knockdown experiments demonstrated an inhibitory effect of ZNF580 on IL-6 expression, consistent with the reporter gene assay and measurements with ZNF580 overexpression experiments in HEK293 cells.

In some experiments, after stimulation of MonoMac6 cells with different LPS concentrations, the total amount of IL6 secreted into the supernatant for 24 h was measured (Fig. 4b, d). At 24 h after stimulation with LPS, ZNF580 tended to be low with a dose-dependent effect of LPS (Fig. 4c). Therefore, increasing levels of secreted IL-6 correlated well with decreasing levels of intracellular ZNF580 (Fig. 4d). Although this does not prove the interaction of ZNF580 and IL-6, it also does not contradict ZNF580-dependent inhibition of IL-6.

We demonstrated the inhibitory effect of ZNF580 on IL-6 in four different approaches. This may agree with the literature, since C2H2-type zinc finger proteins mainly act as suppressors of gene transcription [20] and the suppression of the cytokine IL-8 has already been described for ZNF580 [8]. Others, however, have reported that ZNF580 induces expression of IL-8 in the endothelial like cell line EA.hy926 [9].

One difficulty in interpreting the data is that LPS stimulates the expression of IL-6 but also alters that of ZNF580. This required a closer examination of the time course. Following stimulation with LPS, there was a biphasic response of ZNF580 on the mRNA level (Fig. 5a) followed with some delay by a biphasic response on the protein level (Fig. 5b, c). After two hours of LPS stimulation, mRNA and protein were both increased. After four hours, the protein was still increased while the mRNA was back to control levels or even lower. While the lowest point of mRNA was observed at eight hours, the minimum of the protein level followed at 16 h. After that, the mRNA went up again followed by increasing protein concentrations of ZNF580.

To compare the time course of ZNF580 with IL-6 secretion, the IL-6 secretion rate was calculated (Fig. 5d). After stimulation with LPS, the concentration of ZNF580 protein was increased between 1 h 8 h. Hence, in this early period of LPS stimulation ZNF580 might be part of a negative feedback mechanism that prevents an uncontrolled increase of IL-6. LPS activates NFκB in monocytic cells [14] in a transient manner [18, 19] and at least in EA.hy926 cells NFκB was reported to upregulate ZNF580 [9]. Thus, it seems to be in line with this literature that our study showed elevated levels of ZNF580 after one to eight hours of LPS stimulation.

The reduced ZNF580 concentration 16 h after LPS stimulation was associated with the continued production of IL-6. The increase in ZNF580 from its low point at 16 h to the values at 24 h was accompanied by a decrease in the IL-6 production rate to baseline values. Thus, the low ZNF580 level 16 h after LPS stimulation could disinhibit IL-6 production during the late response to LPS (Fig. 6). The subsequent increase of ZNF580 could eventually stop the production of IL-6. At present, there is no reasonable hypothesis about the pathways that reduce ZNF580.

ZNF580 mRNA was increased in MonoMac6 by LPS after 24 h incubation time. Other mediators/stimulators of inflammation, i.e. fMLP and TNF-α failed to change the relative concentration of ZNF580 mRNA copies after 24 h of incubation time. However, TNF-α- or fMLP-induced effects might be observed at different time points or at different concentrations.

## Conclusions

This study demonstrated a reduced LPS-dependent induction of IL-6 when ZNF580 was elevated. It detected the presence of ZNF580 in monocytes (MonoMac6) where it could thus modulate the production of IL-6. The idea of ZNF580 being a brake on IL-6 production was further supported in MonoMac6 by a negative correlation between these two factors. Moreover, a knock down of ZNF580 facilitated early LPS-induced IL-6 secretion. Therefore, detection of ZNF580 as a suppressor, at least of LPS-induced IL-6 expression, was provided. In addition, ZNF580 was also affected by LPS, indicating an interesting time course similar to a transient regulatory process. Thus, early LPS-increased ZNF580, as shown here, could limit the early LPS-induced peak production of IL-6. LPS-induced reduction of ZNF580 at 16 h could contribute to maintain the production of IL-6 at this later time point. The subsequent increase in ZNF580 could put an end to IL-6 production. In summary, we provide data that ZNF580 is a novel suppressor of IL-6 and that this suppression is modulated in response to LPS stimulation.

## Abbreviations

BSA: Bovine serum albumin
eNOS: Endothelial nitric oxide synthase
FCS: Fetal calf serum
fMLP: Formyl-Methionyl-Leucyl-Phenylalanin
GAPDH: Glyceraldehyde 3-phosphate dehydrogenase
HUVEC: Human endothelial vein endothelial cells
IL-6: Interleukin 6
IL-8: Interleukin 8
LDL: Low density lipoprotein
LPS: Lipopolysaccharide
MMP-2: Matrix metaloprotease 2
mRNA: Messenger ribonucleic acid
PBS: Phosphate buffered saline
PCR: Polymerase chain reaction
PEST: Penicillin/streptomycin
rcf: Relative centrifugal force
TGF-β: Transforming growth factor β
TNF-α: Tumor necrosis factor alpha
VEGF-A: Vascular endothelial growth factor A
ZNF580: Zinc finger protein 580
